# Constraint Therapy with and Without Virtual Reality for Children with Unilateral Cerebral Palsy: A Randomized Trial

**DOI:** 10.3390/children12030283

**Published:** 2025-02-26

**Authors:** Heather Roberts, Nancy J. Clegg, Wayni Wang, Sydney Chapa, Briana Arellano, Madison Trahan, Fabiola Reyes, Mauricio R. Delgado, Sue Ram, Angela Shierk

**Affiliations:** 1 Department of Occupational Therapy, Texas Woman’s University, Denton, TX 76204, USA; wwang@twu.edu (W.W.); schapa2@twu.edu (S.C.); barellano1@twu.edu (B.A.); mtrahan1@twu.edu (M.T.); dram@twu.edu (S.R.); 2 Scottish Rite for Children, Dallas, TX 75219, USA; nancy.clegg@tsrh.org (N.J.C.); fabiola.reyes@tsrh.org (F.R.); mauricio.delgado@tsrh.org (M.R.D.); angela.shierk@tsrh.org (A.S.); 3 Physical Medicine & Rehab Department, University of Texas Southwestern, Dallas, TX 75390, USA; 4 Neurology Department, University of Texas Southwestern, Dallas, TX 75390, USA; 5 Applied Clinical Research Department, University of Texas Southwestern, Dallas, TX 75390, USA

**Keywords:** cerebral palsy, developmental disabilities, healthcare equity

## Abstract

Background/Objectives: Cerebral palsy (CP) is the most common childhood motor disorder, with unilateral cerebral palsy (UCP) presenting with asymmetrical motor function that can cause decreased upper limb function. Constraint-Induced Movement Therapy (CIMT) is an evidence-based intervention that addresses upper limb functional limitations, but further study on combining interventions with CIMT is warranted. Combining CIMT with virtual reality (VR) is hypothesized to enhance engagement and therapeutic outcomes. This study compared the effectiveness of CIMT alone versus CIMT combined with VR (CIMT + VR) in improving upper limb function and occupational performance in children with UCP. Methods: A blinded, randomized, controlled trial included children aged 5–13 years with Manual Ability Classification System (MACS) levels I–III. The participants were randomized into CIMT or CIMT + VR groups and completed a standardized 10-day camp protocol (60 h). Pre-intervention and post-intervention assessments included the Assisting Hand Assessment (AHA) and the Canadian Occupational Performance Measure (COPM). Secondary measures included acceptability ratings of VR devices and fidelity. Results: Thirty-two participants, with a mean age of 9 years and 3 months (3 years 1 month), MACS I = 4, II = 20, and III = 8, completed this study. CIMT and CIMT + VR led to significant improvements in upper limb function, with no statistically significant differences between the groups in bilateral hand use and occupational performance. Conclusions: These findings reinforce the efficacy of CIMT while highlighting the potential of VR to enhance engagement when the child prefers to interact with the technology, underscoring the importance of individualized approaches that consider a child’s preferences and responsiveness to different intervention modalities.

## 1. Introduction

Cerebral palsy (CP) is the most common motor disorder in childhood, affecting approximately 1 in 323 children in the United States [1]. CP is a non-progressive, neurodevelopmental motor disorder caused by brain lesions that develop in the fetal or infant brain and affect movement, posture, coordination, and daily functioning [2,3]. Children with unilateral cerebral palsy (UCP) experience asymmetrical impairments and functional limitations in their upper extremities (UEs) [2,4]. As a result, children with UCP compensate for the lack of functionality in their involved UEs, leading to learned non-use and developmental disregard [4,5]. Developmental disregard is “failure to use the potential motor functions and capacities of the affected arm and hand for spontaneous use in daily life” [6]. The need for an upper limb intervention with practical application is critical to target skills related to the functional limitations and challenges children with UCP commonly face. Children with cerebral palsy (CP) have access to various evidence-based therapies designed to improve upper limb function and participation in daily activities. Bimanual therapy is one of the most widely used interventions, focusing on the coordinated use of both hands through structured practice to enhance functional independence [4,5]. Task-oriented training incorporates meaningful, goal-directed activities to improve motor performance in real-world contexts [7]. Additionally, robot-assisted therapy has emerged as a promising approach, utilizing exoskeletons and sensor-based devices to facilitate movement practice [8]. Other modalities, such as neuromuscular electrical stimulation (NMES) and constraint-based approaches, also play a role in optimizing upper limb function [9,10].

Among these interventions, Constraint-Induced Movement Therapy (CIMT) is a well-established treatment that specifically targets learned non-use by restraining the unaffected limb and encouraging intensive use of the affected limb [11,12]. While CIMT is effective, integrating novel approaches such as virtual reality (VR)-based interventions may further enhance engagement and therapeutic outcomes by providing external multisensory feedback and interactive learning environments [8,13].

Constraint-Induced Movement Therapy (CIMT) is an evidence-based intervention for treating children with UCP. Taub and colleagues first developed Constraint-Induced Movement Therapy (CIMT) to address learned non-use in individuals with neurological impairments [11,12]. The therapy is based on the principle of forced use, in which the unaffected limb is restrained, and the affected limb is intensively engaged in structured activities. CIMT has been widely studied and applied in pediatric rehabilitation, with evidence supporting its ability to drive neuroplasticity and improve functional outcomes in children with UCP [6,9]. The intervention leverages task-specific training and massed practice, reinforcing cortical reorganization and motor skill development [11]. CIMT involves restraining the preferred limb for a specific time and providing structured practice incorporating the concept of “shaping”. Shaping is a strategy wherein tasks are introduced at a rate that promotes success while the task’s difficulty is slowly increased to encourage using the affected limb [14]. Shaping is defined as a structured training method that incrementally increases task complexity based on the child’s performance. This approach is rooted in motor learning theory and aims to maximize functional gains by reinforcing minor improvements over time [9]. Studies indicate that shaping enhances engagement and motivation by maintaining an optimal challenge point, crucial for successful motor rehabilitation [11,12]. CIMT reduces the effects of learned non-use through repetitive tasks that promote cortical reorganization by shaping motor skill development [8]. CIMT is an effective intervention for children with UCP because it encourages the use and skill development of the affected limb compared to no intervention [11,12]. However, using CIMT alone does not fully restore the function of the upper limb in children with CP. As a result, the exploration of combination therapies is warranted to explore additional upper limb therapy interventions that may improve overall effectiveness.

There is limited research that combines CIMT and virtual reality (VR). VR technologies are an upcoming therapeutic tool that has interested therapists who work with children to motivate them during therapeutic sessions. VR can transform repetitive tasks into engaging, functional, and challenging activities, and the difficulty of the tasks can be graded according to ability. Here, VR was defined as a brain–computer interface that provides external feedback to multiple sensory systems while the individual engages in a simulated game or activity [13]. Individuals with neurological impairments have deficits in recognizing and processing internal sensory feedback. Receiving additional external feedback (visual, auditory, kinesthetic) on their performance increases the likelihood of improved performance. Recent VR studies have focused on whole-body movement or just upper or lower body function, but few specifically investigate how VR technology can impact children with UCP and what impact it has on their activities of daily living (ADL) performance [10]. This study investigated the changes following an established intervention, CIMT, to a novel approach of combining CIMT with VR (CIMT + VR).

## 2. Materials and Methods

### 2.1. Study Design

This study was a blinded, randomized, controlled trial designed to compare the effects of Constraint-Induced Movement Therapy (CIMT) alone and CIMT combined with virtual reality (CIMT + VR) on upper limb function and occupational performance in children with unilateral cerebral palsy (UCP). Ethical approval was obtained from the University of Texas Southwestern Institutional Review Board (STU-2021-0271), and the trial was registered on Clinical Trials.gov (NCT06506682). Written informed consent was obtained from all the participants’ guardians, and assent was obtained from children 8 years and older.

### 2.2. Participants

The participants were recruited from a tertiary care center in the Southwest from 2021–2022. The inclusion criteria for the participants included a diagnosis of UCP or a non-progressive brain lesion, injury, or trauma of the developing brain that presents with unilateral upper limb impairment. The participants were classified as GMFCS level I or II and MACS level I–III [14]. All the participants were between 5 and 14 years old and were able to follow directions, complete the assessment protocol, and participate in group activities. All ethnic/racial groups and English-speaking participants were eligible to participate. Any participant with uncontrolled epilepsy or significant visual impairment was excluded from this study. Any patient with severe behavioral problems or asymmetric presentation of upper limb function demonstrating impairments in bilateral upper extremities was also excluded.

A priori power analysis was conducted to determine the minimum sample size. With the alpha set at 0.05, the power set at 0.8, and a moderate-large effect size of 0.30 (f), a total of 24 participants were needed for a repeated-measures analysis of variance (RM ANOVA) (2 groups × 2 time points).

### 2.3. Randomization and Blinding

The participants were randomly assigned by the first author to either the CIMT or CIMT + VR group using a computer-generated randomization sequence from Research Randomizer (https://www.randomizer.org/ (accessed on 7 June 2021)). To ensure allocation concealment, the last author generated and implemented the sequence after baseline assessments were completed each camp year. The assessor scoring the primary outcome measure, the Assisting Hand Assessment, remained blinded to group assignments and the timing of the assessments throughout this study.

### 2.4. Intervention Protocol

Following the established “Pirate Camp” protocol [15], the participants underwent 60 h of intervention (6 h/day over 10 consecutive weekdays). The full pirate camp protocol, including the link to the camp manual, was published by Roberts et al. (2021) [15]. Both groups engaged in activities targeting the affected upper limb’s functional use [15]. The CIMT-only group performed structured, task-specific practice to improve components such as grasp, release, wrist extension, and finger movements. The CIMT + VR group integrated VR-based tasks using devices including Hocoma Armeo^®^Spring Pediatric, Volketswil, Switzerland, Tyromotion Pablo^®^, Graz, Australia, FitMi, Flint Rehab, Irvine, CA, USA, Nintendo Wii^®^, Shenzhen, China, and Parrot Drones, Paris, France. The VR tasks were tailored for engagement and graded difficulty, providing external multisensory feedback (visual, auditory, and kinesthetic).

Hocoma Armeo^®^Spring Pediatric: The Hocoma Armeo^®^Spring Pediatric combines the use of an exoskeleton device with VR technology to practice repetitive movements to improve the functional use of the UE. It can be customized for the range of motion and the amount of gravity assistance the exoskeleton provides the child while playing VR games.

Tyromotion Pablo^®^: The Tyromotion Pablo^®^ Upper Extremity is a sensor-based rehabilitation device. It has built-in strength sensors that measure the hand’s extension and flexion force and interactive therapy games that ensure target-oriented repetitive therapy.

FitMi: FitMi is a neurorehabilitation device that provides visual feedback on motor performance as the individual interacts with wireless switches. Patients use two wireless buttons to interact with therapeutic exercise apps on a tablet. Each button has sensors that track the patient’s movements to provide real-time feedback.

Nintendo^®^ Wii: The Nintendo^®^ Wii is a video game console with a handheld pointing device that detects movement in three directions. The system requires the player to hold the controller in the hand while moving the UE.

Parrot Drones: Parrot Drones are quadcopter mini drones that can be flown indoors and outdoors. The drones are controlled by either a remote control or a smart device that targets isolated finger and thumb movements.

Both groups engaged in 60 h of intervention (Monday–Friday, 6 h per day for 10 days). Approximately 50 h were CIMT, and 10 h were focused on bimanual skills. The CIMT + VR group engaged in 16 of the 60 h using VR technology, while the CIMT-only group engaged in task-specific practice aimed at improving the same components of upper limb function as the CIMT + VR group (use of the assisting hand, stabilizing/holds, reach, grasp, release, supination, wrist extension, finger movements, thumb opposition, grip, and pinch strength). The study protocol and outcome measures remained consistent throughout the trial.

### 2.5. Outcome Measures

The primary outcomes were bimanual hand skills, assessed with the Assisting Hand Assessment (AHA), and occupational performance, measured with the Canadian Occupational Performance Measure (COPM). These assessments were conducted pre-intervention and post-intervention. The secondary outcomes included fidelity of intervention, and the acceptability of the VR equipment was rated daily on a 5-point Likert scale, with participants providing feedback on their favorite and least favorite devices and any discomfort.

Assisting Hand Assessment (AHA): The AHA is a valid and reliable 22-item measure assessing the affected hand in bimanual activities for children with CP or obstetric brachial plexus palsy [14].

Canadian Occupational Performance Measure (COPM): The COPM is an individualized, client-centered outcome measure designed for occupational therapists to detect changes in the self-perception of occupational performance over time. It is a reliable tool for clients with various disabilities and developmental levels [16].

Acceptability: Following each VR training session, the CIMT + VR group rated the acceptability of each VR equipment utilized using a 5-point Likert scale. The participants also wrote down their favorite and least favorite VR technology, which devices were too easy or hard to use, and whether any devices were uncomfortable. The participants were also allowed to provide comments about their selections.

Fidelity: The study personnel completed fidelity checks throughout the 10-day camp using a modified version of the MR3 Cycle [7]. The MR3 Cycle guides learning progression by shaping supports and demands toward targeted motor and functional outcomes through movement, reinforcement, repetition, and refinement [7] 14. The fidelity of this study was measured across five domains based on observations of both the child and the interventionist during the intervention, including (1) the interventionist’s choice of activity, (2) interaction and engagement with the participant, (3) guidance and modeling of the upper limb domain, (4) reinforcement and feedback, (5) repetition of practice, and (6) shaping. The fidelity ratings for each domain were based on a three-point scale. A score of three indicated the intervention met high standards and expectations, two represented acceptable standards and expectations, one suggested the intervention did not meet standards and expectations.

### 2.6. Statistical Analysis

Descriptive statistics were performed to report sample characteristics. A series of repeated-measure ANOVAs were used to evaluate within- and between-group differences for primary and secondary outcomes. Independent *t*-tests were conducted to compare the change scores (Δ) between the two groups. Statistical significance was set at *p* < 0.05, with analyses performed using SPSS version 30. Missing data were addressed by applying pairwise deletion methods. After year one, interim analyses were performed to ensure protocol adherence and accuracy.

### 2.7. Data and Materials

All the study materials, including protocols, randomization sequences, and raw data, are available upon request from the corresponding author.

## 3. Results

### 3.1. Participant Characteristics

**Figure 1:**
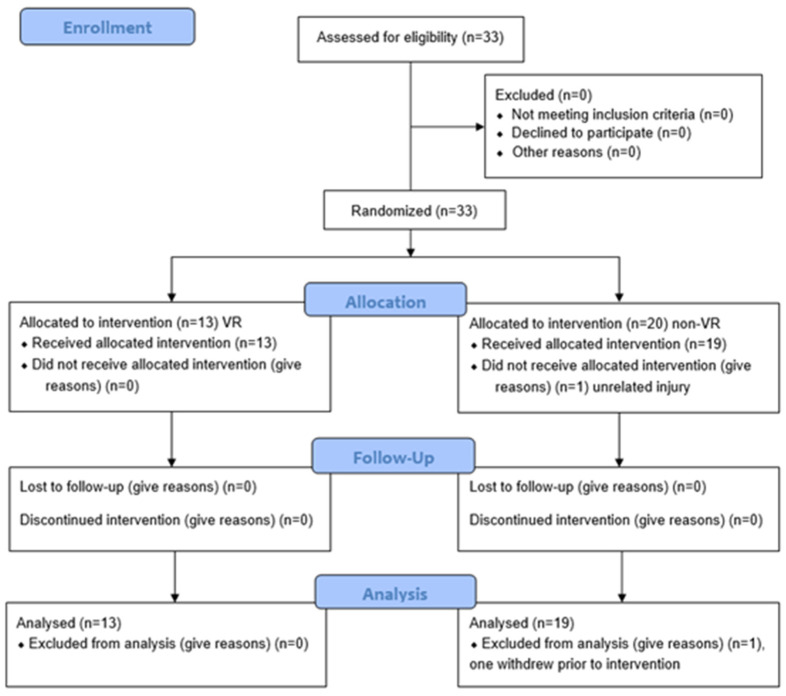
CONSORT flow diagram.

Thirty-three datasets from 23 unique participants were analyzed, including 9 who completed the intervention twice over the two years, with re-randomization for the second year (Figure 1). Sufficient power was achieved. Participants ranged in age from 5 to 14 years (mean = 9 years, 3 months), with Manual Ability Classification System (MACS) levels distributed as follows: level I (*n* = 4), level II (*n* = 20), and level III (*n* = 8). Gender representation included 13 males and 19 females (Table 1).

### 3.2. Intervention Efficacy

Both Constraint-Induced Movement Therapy (CIMT) alone and CIMT combined with virtual reality (CIMT + VR) demonstrated statistically significant improvements in bimanual hand function and occupational performance from pre-intervention to post-intervention.

### 3.3. Assisting Hand Assessment (AHA)

**Figure 2:**
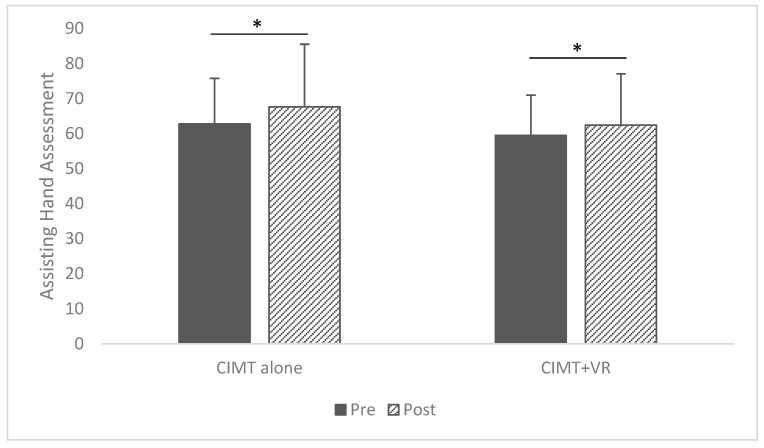
Pre- and post-intervention Assisting Hand Assessment mean scores. * Statistical Significance.

The results demonstrated a significant time effect (*F* = 18.57, *p* < 0.001) with a large effect size of 0.39 (Partial *η*^2^). Still, the interaction effect and group main effect were not significant, with small effect sizes in the AHA. Specifically, in Figure 2, CIMT improved the scores from the pretest (*M* = 62.74, *SD* = 13.06) to the posttest (*M* = 67.63, *SD* = 11.49), *p* < 0.001. The CIMT with VR group also increased AHA scores from the pretest (*M* = 59.50, *SD* = 17.89) to the posttest (*M* = 62.42, *SD* = 14.65), *p* = 0.049. Change scores (Δ) were computed from the pretest to the posttest, but no significant difference in the changes was identified between CIMT and CIMT + VR (*p* = 0.284).

**Figure 3:**
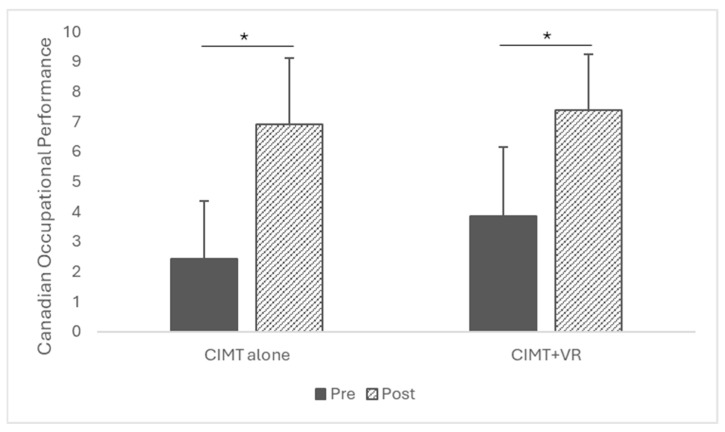
Pre- and post-intervention COPM performance mean scores. * Statistical Significance.

Canadian Occupational Performance Measure (COPM): There was no significant interaction effect (time × group: *F* = 1.02, *p* = 0.322). However, a significant time effect was found on the overall sample (*F* = 76.51, *p* < 0.001) with a very large effect size (Partial *η*^2^ = 0.73). Regardless of the intervention group, the performance was significantly improved from pretest to posttest (Figure 3). In CIMT alone, the score significantly increased from pretest (*M* = 2.44, *SD* = 1.93) to posttest (*M* = 6.90, *SD* = 2.23), *p* < 0.001. In the CIMT with VR group, the performance scores were also higher at the posttest (*M* = 7.38, *SD* = 1.86) as compared to the pretest (*M* = 3.84, *SD* = 2.33), *p* < 0.001. Even though there was no significant difference between CIMT and CIMT + VR on the overall sample (*F* = 2.23, *p* = 0.146), the effect size (Partial *η*^2^ = 0.07) indicates a moderate magnitude of differences between the groups. The change scores for performance were computed from pretest to posttest, but the CIMT and CIMT + VR groups did not differ on the performance improvement (*p* = 0.322) with a small–moderate effect size (*d* = 0.38).

### 3.4. Satisfaction

**Figure 4:**
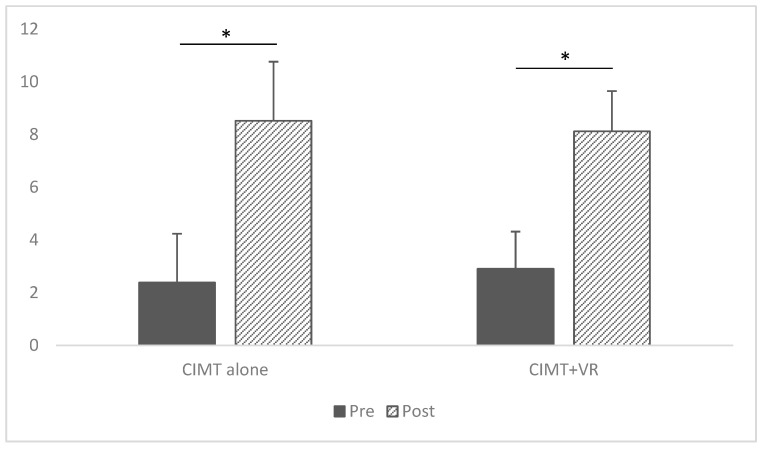
Pre- and post-intervention COPM satisfaction mean scores.* Statistical Significance.

Like the above findings, satisfaction was significantly improved from pretest to posttest on both CIMT alone and CIMT + VR, suggesting the feasibility of both intervention strategies. As shown in Figure 4, the satisfaction scores for CIMT + VR rose from an average of 2.90 to 8.12 (*p* < 0.001); the satisfaction scores for CIMT alone rose from 2.38 to 8.52 (*p* < 0.001). Change scores were computed from the pretest to the posttest for satisfaction, but no significant difference was found between CIMT and CIMT + VR (*p* = 0.362).

### 3.5. Secondary Outcomes

#### Acceptability

The participant feedback on the VR devices revealed high enjoyment ratings for the Nintendo^®^ Wii (80% rated as enjoyable) and drones (82%). In contrast, FitMi and Hocoma Armeo^®^Spring received mixed feedback, with approximately 30% of participants rating them as not enjoyable and 19% as neutral. Pablo^®^, FitMi, and Armeo^®^ also received around 50% of responses indicating enjoyment.

The participants frequently selected the Wii (39.82%) and the drone (32.74%) as their favorite devices, while the FitMi (35.35%), Armeo^®^ (28.28%), and Pablo^®^ (23.23%) were most often reported as least favorite. Some participants reported discomfort with Pablo^®^ (grip, grasp, and weight), Armeo^®^ (setup and adjustments), FitMi (tapping), and Wii (controller size). Interventionists provided adjustments, breaks, or alternative game options when discomfort was reported. No adverse events occurred during the trial.

### 3.6. Fidelity of Implementation

Across all six domains, the fidelity ratings fell below standards and expectations less than 1% of the time. Ratings at the highest level, indicating high standards and expectations, ranged from 55% to 85% across domains (Table 2). The domains with the lowest percentages of high ratings were guidance modeling and repetition, while interaction and engagement received the highest percentage of high ratings.

## 4. Discussion

This study evaluated the efficacy of CIMT alone compared to CIMT + VR for improving upper extremity function in children with UCP. Both interventions demonstrated statistically significant improvements in bimanual hand function and occupational performance, consistent with the existing literature on intensive therapy approaches for UCP [11,12]. However, the two groups had no statistically significant differences, with the CIMT-alone group showing slightly more significant mean changes. These findings provide insight into integrating VR into established therapeutic protocols and highlight the importance of considering both efficacy and individual preferences.

### 4.1. Comparison with Existing Research

The findings align with prior research that underscores the effectiveness of CIMT in reducing developmental disregard and promoting cortical reorganization in children with UCP [6,9,11]. CIMT continues to demonstrate its ability to drive functional improvements in the affected limb as an intervention grounded in repetitive practice and task shaping. The addition of VR is based on the hypothesis that external feedback and increased engagement could address attention and motivation challenges, though its impact on overall therapeutic outcomes remains uncertain [8,13].

Despite the theoretical advantages of VR, this study’s results did not reveal significant added benefits for CIMT + VR compared with CIMT alone. This outcome aligns with recent meta-analyses suggesting that while VR interventions are promising, their effects on motor outcomes may be comparable to those of traditional evidence-based therapies [7,10]. The lack of statistically significant differences between the groups could be due to both interventions being engaging, with participants in both groups demonstrating high compliance in practicing the tasks. These findings highlight the efficacy of CIMT as a standalone intervention, suggesting that it is better that the choice between CIMT and CIMT + VR be guided by the child’s individual preferences rather than the child being assigned an intervention.

The importance of personalized therapy approaches is increasingly recognized in pediatric rehabilitation, particularly as technological advancements and AI-driven interventions enable more tailored treatment strategies. The research suggests that incorporating child-specific preferences, motivation, and engagement levels into therapy can enhance adherence and optimize functional outcomes [8,11].

In the present study, individual differences in engagement were evident, with children expressing varying levels of enjoyment toward different VR devices. This aligns with the existing literature that underscores intrinsic motivation as a key factor in motor learning and rehabilitation success [10,13]. Technologies such as AI-driven adaptive therapy programs have emerged as promising tools to personalize rehabilitation further, allowing interventions to be dynamically adjusted based on real-time performance data and user feedback [7]. Future research should explore adaptive and AI-integrated rehabilitation models that tailor task difficulty, feedback delivery, and intervention selection based on a child’s unique cognitive, motor, and sensory factors. Such individualized therapy frameworks have the potential to maximize treatment adherence, engagement, and long-term functional improvements, paving the way for more effective and child-centered rehabilitation approaches.

### 4.2. Practical Implications

The slight advantage observed in the CIMT-alone group may reflect the benefits of uninterrupted task-specific practice, a cornerstone of CIMT’s effectiveness [11,12]. In contrast, while VR introduces a novel and engaging element, its complexity and novelty may limit its effectiveness for some children [8,10].

The acceptability data highlight the importance of tailoring interventions to individual preferences. Devices like the Nintendo^®^ Wii and drones, rated highly for enjoyment, demonstrate the potential of simple, intuitive VR tools for promoting engagement. However, the mixed feedback on more complex devices underscores the need for careful device selection based on ergonomic and developmental considerations [10,13].

### 4.3. Implications for Future Research

This study contributes to the growing body of literature exploring technology integration in pediatric rehabilitation. Future research should address the following areas:

- Long-term outcomes: investigating the sustainability of gains achieved through CIMT + VR in everyday functional activities and participation [7,8].
- Customizing VR applications: further exploration of the underlying motivation and personal desires driving engagement in virtual reality activities is essential to understanding how individualized preferences and intrinsic interests influence therapeutic adherence and outcomes [10].
- Multi-center trials: expanding research across diverse populations to enhance generalizability and examine the scalability of CIMT + VR [8,11].
- Cost-effectiveness analysis: evaluating the feasibility and affordability of VR interventions in clinical and home settings [8].

### 4.4. Broader Context

Integrating VR into pediatric rehabilitation holds immense potential for transforming therapy into an engaging, child-friendly experience. However, as this study shows, the choice to incorporate VR should be guided by clinical judgment, child-specific factors, and the availability of resources. While VR offers a unique opportunity to diversify therapeutic tools, the foundational principles of intensive, task-specific practice remain critical for driving meaningful outcomes. Studies have shown that parents may hesitate to adopt technology-based interventions, citing concerns regarding effectiveness, screen time, or reduced hands-on approaches [8,13]. Socioeconomic and cultural factors may also influence the acceptance of VR [10]. Addressing these concerns requires clear communication on how VR complements traditional therapy, enhances engagement, and supports real-world skill transfer [7]. Hybrid models combining VR with structured hands-on activities may further increase family acceptance and improve adherence.

### 4.5. Limitations

There were a few limitations in this study. First, the generalizability may have been impacted as the participants were recruited from one geographical location. Second, the current research using VR is varied compared to other forms of evidence-based protocols. There is a lack of previous research studies in this area, therefore limiting the scope of this research. Lastly, replicability may not be possible if a researcher does not have the specific types of VR used in this study. It may be possible to replicate this study using other forms of VR that achieve the same goals as the technology used in this study.

## 5. Conclusions

This study demonstrates that both Constraint-Induced Movement Therapy (CIMT) and CIMT combined with virtual reality (CIMT + VR) are effective interventions for improving upper extremity function and occupational performance in children with unilateral cerebral palsy (UCP). While both approaches yielded significant improvements, no statistically significant differences existed between the groups. CIMT alone showed slightly higher mean changes in performance outcomes. These findings highlight the continued efficacy of CIMT as a standalone intervention while emphasizing the potential of VR to enhance engagement and therapy adherence. The integration of VR should be guided by individual preferences and the child’s specific needs. Future research should explore long-term outcomes, broader implementation, and personalized applications of VR technologies to maximize their therapeutic impact. Overall, this study underscores the value of combining innovative technologies with evidence-based practices to address the diverse needs of children with UCP when working to improve their upper limb function.

## Figures and Tables

**Table 1:** Participant demographics.

	VR (*n* = 13)	CIMT-Only (*n* = 19)	Combined (*n* = 32)
Gender			
Male	6	7	13
Female	7	12	19
Age	10 yr 3 mo (3 yr 0 mo)	8 yr 7 mo (3 yr 1 mo)	9 yr 3 mo (1 yr 3 mo)
MACS			
I	2	2	4
II	8	12	20
III	3	5	8

Note. Virtual reality (VR), Constraint-Induced Movement Therapy (CIMT), Manual Ability Classification System (MACS).

**Table 2:** Fidelity responses.

Fidelity Ratings	Choice of Activities	Interaction and Engagement	Guidance Modeling	Reinforcement	Repetition	Shaping
**1**	<1%	<1%	0.01%	0.01%	0.01%	0.01%
**2**	24.50%	13.90%	37.70%	15.20%	38.40%	36.40%
**3**	74.80%	85.40%	60.90%	82.70%	59.60%	62.20%

## Data Availability

The data supporting this study’s findings are not publicly available due to privacy and ethical restrictions involving pediatric participants. Data may be made available upon reasonable request to the corresponding author, subject to Institutional Review Board (IRB) approval and adherence to confidentiality agreements.

## Abbreviations

The following abbreviations are used in this manuscript:

CP	Cerebral palsy
UE	Upper extremity
AHA	Assisting Hand Assessment
UCP	Unilateral cerebral palsy
CIMT	Constraint-Induced Movement Therapy
MACS	Manual Ability Classification System
GMFCS	Gross Motor Function Classification System
VR	Virtual reality
